# Supplementary material for: Snapshot of the Phylogenetic Relationships among Avian Poxviruses Circulating in Portugal between 2017 and 2023
Source: Vet Sci. 2023 Dec 7;10(12):693. doi: 10.3390/vetsci10120693 (PMC10747575; doi:10.3390/vetsci10120693)
Supplement: Supplementary file 1 [file vetsci-10-00693-s001.zip › vetsci-2658986-Supplementary.pdf]

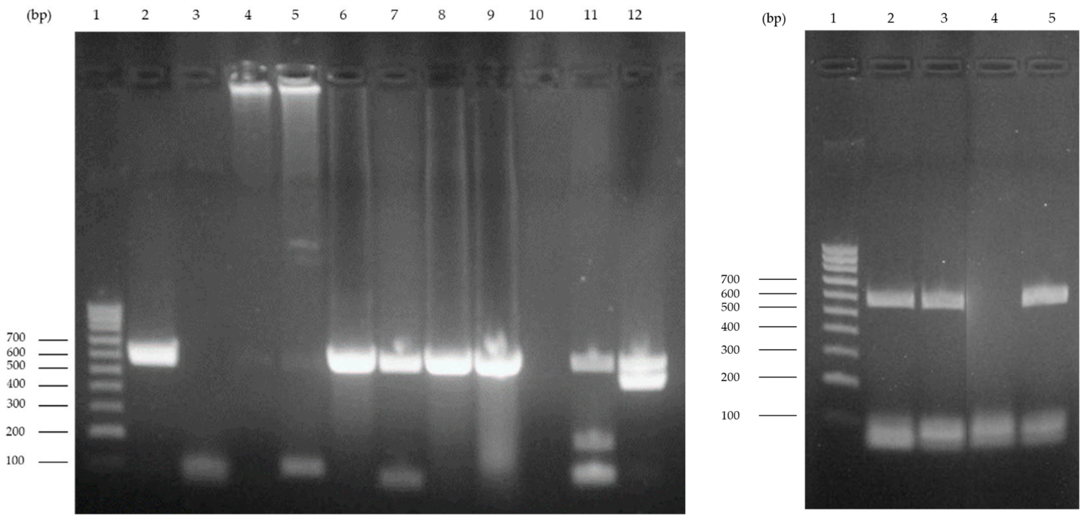

(**a**) (**b**)

Figure S1. A 1% agarose gel electrophoresis of PCR amplification of the 578 bp P4b gene fragment. (**a**) Lane 1 corresponds to the molecular weight marker, NZYDNA Ladder V (NZYTech, Portugal). Lanes 2 to 9 correspond to the amplification of samples 24569-17 (flamingo), 11612-19 (puffin), 37026-19 (canary), 03779-20 (canary), 04482-20 (chicken), 16735-20 (blackbird), P-08508-21 (chicken), and 23049-18 (chicken), respectively. Lane 10 is the negative control. Lanes 11 and 12 correspond to the amplification of samples 37026-19 (canary) and 03779-20 (canary) obtained from DNA previously extracted. (**b**) Lane 1 corresponds to the molecular weight marker, NZYDNA Ladder V. Lanes 2 and 3 correspond to sample 11612-19 (puffin) from different extraction reactions. Lanes 4 and 5 are the negative and positive controls, respectively.
